# Clinical glycomics for the diagnosis of congenital disorders of glycosylation

**DOI:** 10.1007/s10545-018-0144-9

**Published:** 2018-03-01

**Authors:** Nurulamin Abu Bakar, Dirk J. Lefeber, Monique van Scherpenzeel

**Affiliations:** 1 Translational Metabolic Laboratory, Donders Institute for Brain, Cognition, and Behavior, Radboud University Medical Center, Geert Grooteplein 10, Nijmegen, 6525 DA The Netherlands; 2 Department of Neurology, Donders Institute for Brain, Cognition, and Behavior, Radboud University Medical Center, Nijmegen, The Netherlands

## Abstract

Clinical glycomics comprises a spectrum of different analytical methodologies to analyze glycan structures, which provides insights into the mechanisms of glycosylation. Within clinical diagnostics, glycomics serves as a functional readout of genetic variants, and can form a basis for therapy development, as was described for PGM1-CDG. Integration of glycomics with genomics has resulted in the elucidation of previously unknown disorders of glycosylation, namely CCDC115-CDG, TMEM199-CDG, ATP6AP1-CDG, MAN1B1-CDG, and PGM1-CDG. This review provides an introduction into protein glycosylation and presents the different glycomics methodologies ranging from gel electrophoresis to mass spectrometry (MS) and from free glycans to intact glycoproteins. The role of glycomics in the diagnosis of congenital disorders of glycosylation (CDG) is presented, including a diagnostic flow chart and an overview of glycomics data of known CDG subtypes. The review ends with some future perspectives, showing upcoming technologies as system wide mapping of the N- and O-glycoproteome, intact glycoprotein profiling and analysis of sugar metabolism. These new advances will provide additional insights and opportunities to develop personalized therapy. This is especially true for inborn errors of metabolism, which are amenable to causal therapy, because interventions through supplementation therapy can directly target the pathogenesis at the molecular level.

## Introduction

Technological advances in science are creating a revolution in the world of clinical diagnostics for rare metabolic disorders. In many cases, establishing a diagnosis via the traditional care route is a complex, lengthy process involving multiple consultations by various clinical specialists. The fact that an omics technique greatly reduces this turnaround time and increases the diagnostic yield has recently been shown for a cohort of 150 patients presenting with complex neurological disorders of suspected genetic origin. They compared the number of solved cases applying either whole exome sequencing (WES) alone (29.3%) or the standard care pathway (7.3%). This improvement in diagnostic yield is significant, without increasing costs compared to the standard diagnostic trajectory (Vissers et al 2017). However, the percentage of solved cases of about 30% is still quite low, which is likely caused by either the lack of sequence coverage of the variant, by disease causes outside the coding sequences, or the presence of too many “variants of unknown significance”. In this light, the important connection of genomics with functional -omics methodologies in the diagnosis of metabolic disorders is recognized more and more. For example, a combination of WES and deep clinical phenotyping was applied to 41 patients with intellectual developmental disorder and unexplained metabolic abnormalities, which resulted in a diagnosis for 28 patients (68%), and a test for targeted intervention on 18 patients (44%) (Van Karnebeek et al 2016). For congenital disorders of glycosylation (CDG), many cases were unsolved until the inclusion of glycomics into clinical practice, to present the functional defect (Jansen et al 2016a; Jansen et al 2016b; Jansen et al 2016c; Van Damme et al 2016; Van Scherpenzeel et al 2014; Tegtmeyer et al 2014; Carss et al 2013; Iqbal et al 2013). The most important advantage of integrating functional omics with genomics in the field of inherited metabolic disorders is the opportunities for therapy, which do arise from insights into functional, biochemical pathways. Initial evidence was published for PGM1-CDG (Tegtmeyer et al 2014; Wong et al 2017). This review discusses glycomics, its role in CDG diagnostics, presents different methodologies, and ends with future perspectives, in which the horizon of the diagnostic laboratory needs to be broadened to functionally understand new genetic defects.

## Introduction in glycosylation

Protein glycosylation is considered to be the most common post-translational modification and is ubiquitously present (Moremen et al 2012). Glycosylation is a non-template driven process involving multiple competing enzymes (e.g., glycosidases and glycosyltransferases) in the endoplasmic reticulum (ER) and the Golgi apparatus (GA) as glycoproteins traffic and mature through the secretory pathway. Therefore, glycan structures are highly diverse, with multiple possibilities for branching and linkage (micro-heterogeneity) and differences in site occupancy (macro-heterogeneity). Glycans are known to have many important biological functions, such as cell-cell, macromolecular (e.g., antibody) and pathogen interactions, protein secretion, protein signaling, and protein folding (Moremen et al 2012; Defaus et al 2014).

Glycosylation changes have been identified in various diseases, ranging from monogenetic inherited disorders such as CDG (Jaeken 2011) to multiple types of malignancies; e.g., ovarian (Leiserowitz et al 2007), colon (Sethi et al 2014) and breast cancer (Lee et al 2014), but also observed in other pathological situations, such as cirrhosis, hepatitis, and neurodegenerative diseases such as Alzheimer’s (Palmigiano et al 2016) and Parkinson disease (Russell et al 2017). Therefore, the analysis of glycosylation in complex biological matrices, which is called glycomics, has become one of the popular -omics after the era of genomics and proteomics, especially for biomarker discovery, treatment monitoring, and also to understand disease mechanisms. This has become more relevant and promising since the majority of tumor biomarkers endorsed by the Food and Drug Administration (FDA) are glycoproteins (Fuzery et al 2013). Most of the markers are only judged based on their expression level. However, for two glycoproteins, glycosylation is included as a biomarker in routine clinical diagnostics, namely carbohydrate-deficient transferrin for the detection of CDG and alcohol abuse, and fucosylated serum alpha-fetoprotein (AFP-L3) for the early diagnosis of hepatocellular carcinoma (Li et al 2001; Leerapun et al 2007). It was shown that by including glycosylation in the analysis, specificity and sensitivity were increased over the native alpha-fetoprotein, and intact transferrin mass spectrometry resulted in the direct diagnosis of several CDG-II subtypes (van Scherpenzeel et al 2015).

**Fig. 1:**
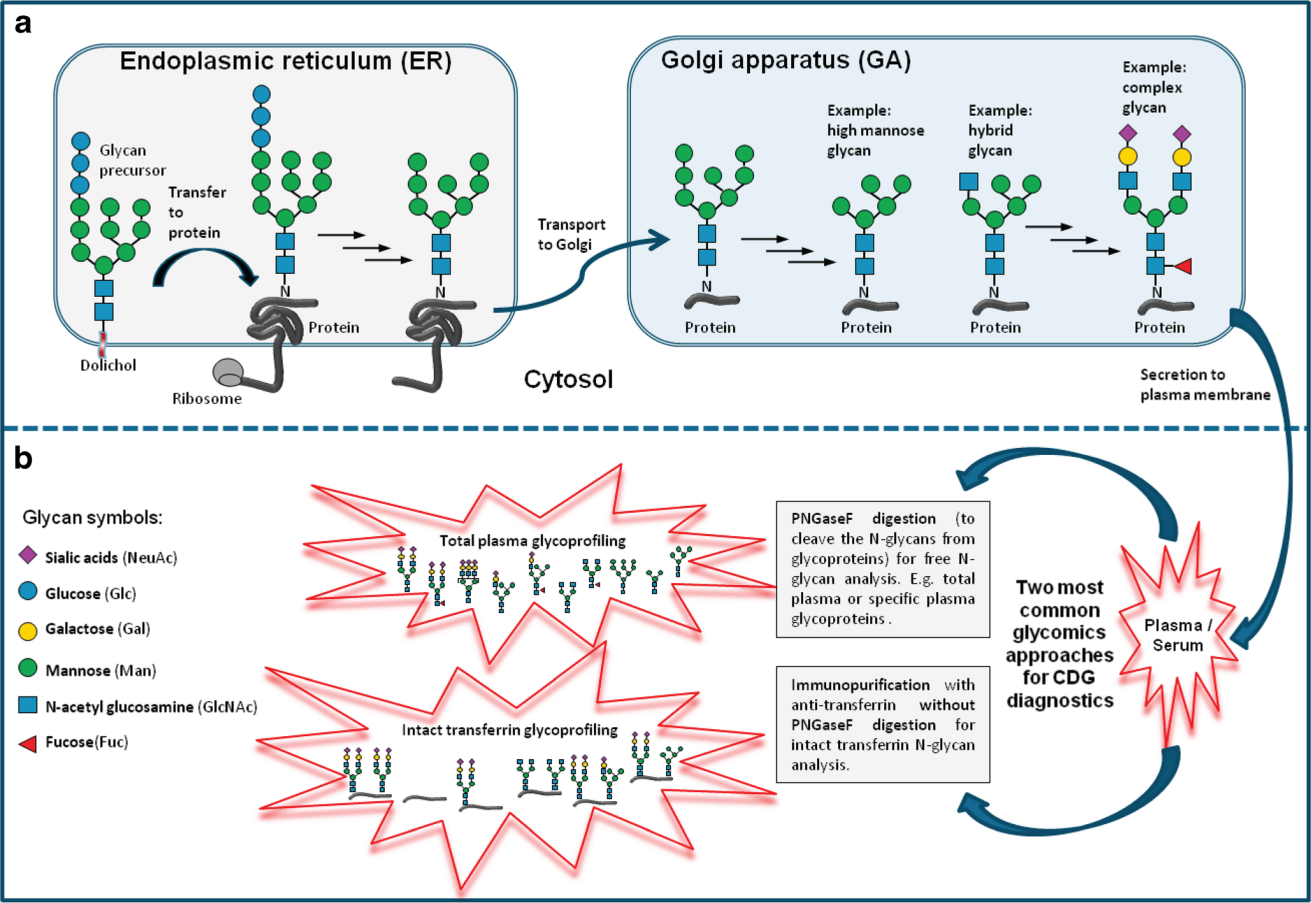
Schematic representation of the process of protein N-glycosylation, the different types of N-glycans (a) and their analysis by mass spectrometry in CDG (b) **(a)** The process of N-glycosylation is started when a glycan precursor is assembled by sequential addition of monosaccharides onto the lipid anchor dolichol in the membrane of the ER. The glycan precursor containing 14 monosaccharides (Glc_3_-Man_9_-GlcNAc_2_) is then transferred en bloc to a specific asparagine residue (N) within the consensus sequence N-X-S/T (X = any amino acid except proline, S = serine, T = threonine) in the nascent peptide chain of a protein (e.g., transferrin) being synthesized by a ribosome. After several glucose trimming by ER glucosidases, the glycoprotein is transported to the GA, where the glycans are modified in multiple steps through the action of various glycosidases (trimming) and glycosyltransferases (prolonging). All N-glycans share the common core structure of Man_3_-GlcNAc_2_, and are classified into high mannose glycan (only Man residue attached to the core), complex glycan (only GlcNAc residues are attached to the core) and hybrid glycan (combination of Man and GlcNAc residues are attached to the core). **(b)** The two most common plasma glycomics approaches for CDG characterization are free N-glycans profiling (glycans released from whole or specific glycoproteins by PNGAseF digestion; e.g., total plasma glycoprofiling) and intact protein glycoprofiling (immunopurification protocol without PNGAseF digestion)

There are three types of protein glycosylation in mammalian cells, namely N-, O-, and C-linked glycosylation each having their own subclasses (Moremen et al 2012). The first two are the most common types of protein glycosylation in human cells (Wang et al 2014), and are therefore also the most studied and best characterized types. This review on clinical glycomics will be restricted to these two most common types. N-linked glycans are attached to the polypeptide via amide linkages to asparagine (Asn) side chains, while O-glycans are attached through glycosidic linkages to side chains of serine (Ser) or threonine (Thr). N-glycans consist of several monosaccharides, such as *N*-acetylglucosamine (GlcNAc), mannose (Man), fucose (Fuc), galactose (Gal), glucose (Glc), and sialic acid (Sia), which are built in a specific order (Cummings 2009). Briefly, N-glycans are assembled in the ER before being further processed and modified in the GA producing three types of N*-*glycans, namely complex, hybrid, and high mannose (Fig. 1a).

In contrast to N-glycosylation, O-glycosylation is assembled directly onto serine or threonine residues without pre-assembly. Mucin type O-glycans are the most well-known type of O-glycans with *N*-acetylgalactosamine (GalNAc) at the reducing end. Another large group of O-glycans are the glycosaminoglycans (GAGs) on proteoglycans. GAGs are long, unbranched carbohydrates containing repeated GalNAc or GlcNAc residues combined with glucuronic acid (GlcA) or Gal residues. The other five types of O-glycosylation are O-linked GlcNAc, -Gal, −Man, −Glc, and -Fuc. In all of these classes, one or more genetic deficiencies have been identified (Wopereis et al 2006, Hennet 2012, Freeze et al 2014).

## Clinical glycomics methodologies

For protein N-glycan analysis, the sample preparation usually starts with the addition of the enzyme *N*-glycosidase F (PNGaseF), to cleave the N-glycans from the proteins. The method is applicable to purified proteins, as well as on complex biological samples, such as blood, urine, and CSF. In addition to the analysis of protein-released N-glycans, advances in technology allow the analysis of intact glycoproteins, which is fast, robust, does not require enzymatic digestion, and enables high-throughput analysis (Fig. 1b).

Traditionally, derivatization is the common step after releasing N-glycans from proteins, because this type of modification increases the sensitivity of glycan detection as native N-glycans have no significant ultraviolet (UV) absorbance. One could make use of the single reactive carbonyl group at the reducing end of the glycan and perform reductive amination with UV or fluorescent tags, such as 2-aminobenzoic acid (2-AA), 2-aminobenzamide (2-AB), and 2-aminopyridine (2-AP) (Ruhaak et al 2010; Pabst et al 2009), or permethylate glycans (Ciucanu and Kerek 1984; Kang et al 2008), thereby replacing all the hydroxyl groups with methyl ethers. Recently, a method for sialic acid esterification was reported, which not only enhances the stability of acidic glycans during analysis, but also distinguishes alpha2,3 and alpha2,6 sialic acid linkage (Wheeler et al 2009; Reiding et al 2014). Disadvantages of derivatization steps are that they could be incomplete, which creates a biased analysis, as well as the need for additional purification procedures which can cause sample loss (Pabst and Altmann 2011).

Several electrophoretic and chromatographic techniques have been established to separate complex N-glycan mixtures, such as capillary electrophoresis, ion exchange chromatography, hydrophilic interaction liquid chromatography (HILIC), liquid chromatography (LC), and porous graphitized carbon (PGC) (Melmer et al 2011). Recently, PGC-LC-MS has emerged as a popular platform to efficiently separate native glycans without derivatization steps which greatly reduced turnaround time of glycomics sample preparation and enabled the separation of isomeric N-glycans (alpha and beta anomers). Native N-glycan analysis using the PGC-LC approach has been successfully applied for glycan biomarker discovery in ovarian cancer (Hua et al 2013), colorectal cancer (Sethi et al 2015), and lung cancer (Ruhaak et al 2016).

Electrospray ionization (ESI) and matrix assisted laser desorption ionization (MALDI) are the two most common ionization techniques for MS glycan analysis. Both ionization techniques are very sensitive to analyze proteins, peptides, glycans, and lipids as low as picomolar concentrations. ESI is considered as a soft ionization technique, and is able to create multiple charged ions for biomolecules, such as proteins and peptides, which enables these large molecules to be analyzed within the mass over charge ratio (m/z) ranges of the instrument. When coupled to LC and including standards for calibration, ESI is more reliable for quantitative analysis than MALDI (El-Aneed et al 2009; Sturiale et al 2011).

In comparison to ESI, MALDI has the advantages of being robust, fast, and easy to operate. Additionally, MALDI allows analyzing the acidic N-glycans in relative quantities compared to the neutral glycans when derivatization steps, such as permethylation and ethyl esterification, were performed prior to MS to neutralize and stabilize sialic acids. Fourier transform (FT-) and time of flight (TOF)-MS are currently the detectors with the highest resolution (Wuhrer et al 2004; Pabst and Altmann 2008). The second might be preferable considering its capability to analyze large molecules like intact proteins, and the high maintenance costs of FT detectors.

Unfortunately, there is no general O-glycosidase available for enzymatic release of all species of O-glycans. For O-glycan profiling, chemical release by hydrazinolysis or (reductive) beta-elimination needs to be performed, which are harsh methods that always yield some side products. The reductive beta-elimination is the most clean and therefore the most commonly used method, since the simultaneous reduction of the terminal sugar prevents the peeling of the glycan due to the alkaline conditions (degradation from its reducing end) (Thaysen-Andersen and Packer 2014). There are few methods published to simultaneously profile plasma N- and O-linked glycosylation of CDG patients (Faid et al 2007; Xia et al 2013). Because of their limited applicability so far, congenital disorders in the biosynthesis of O-glycans have been identified by genetic approaches. However, for the mucin type O-glycans, intact apolipoprotein C-III (Apo C-III) profiling is available in a diagnostic setting (Wopereis et al 2003). One way to circumvent the need to use harsh conditions to cleave O-glycans is to analyze native glycopeptides, with the additional advantage of keeping the information on the attachment site intact (Hoffmann et al 2016). System-wide mapping of the N- and O-glycoproteome is envisioned in a good review of Thaysen-Andersen and Packer in 2014 (Thaysen-Andersen and Packer 2014). This new frontier in proteomics has the merits of high-resolution MS, complementary fragmentation techniques, and bioinformatic tools.

## Application of clinical glycomics for CDG diagnostics

CDG are a group of genetic defects with abnormal glycosylation of proteins, lipids or both. According to the current guidelines (Jaeken et al 2009), CDG is classified into: protein N-glycosylation defects, protein O-glycosylation defects, glycosphingolipid and glycosylphosphatidylinositol anchor glycosylation defects, and multiple glycosylation pathway defects. Currently, 105 distinct types of CDG have been reported (Jaeken and Peanne 2017; Peanne et al 2017). However, with the emergence of next generation genomics technologies, there were also genes identified that do not directly involve the glycosylation biosynthesis pathway, for example defects in nucleotide sugar transport, defects of vesicular transport, defects in O-mannosylation, O-GlcNAcylation or defects in dolichol biosynthesis, which lead to abnormal protein glycosylation (Hennet 2012). CDG are commonly classified by the localization of the genetic defect (Jaeken et al 2009), in which CDG type 1 (CDG-I) refers to deficient synthesis of the precursor glycan in the ER, also including defects in the cytosol (e.g., PMM2- and PMI-CDG) and the transfer of the LLO to the protein, including various OST defects (e.g., DDOST, SST3A, and SST3B). CDG type 2 (CDG-2) occurs in the GA resulting in modified glycans on the glycoprotein (Lefeber et al 2011). Since the majority of these glycosylation disorders showed a defect in the biosynthetic pathway of protein N-glycosylation (Freeze et al 2014), the analysis of plasma N-glycans using MS plays a significant role in CDG research and diagnostics. The two most common clinical glycomics approaches for CDG characterization are global (N-glycans released from whole serum or plasma glycoproteins, further abbreviated as plasma glycomics) and protein-specific (e.g., intact transferrin MS & Apo C-III MS) glycoprofiling (Fig. 1).

The first application of glycomics was ESI-MS for CDG-I characterization. Transferrin profiles using ESI-MS on PMM2-CDG patients clearly showed two abnormal peaks corresponding to lack of one and both complete glycans (Wada et al 1992a; Wada et al 1992b). ESI-MS of transferrin became more ‘mature’ in 2001, when coupled to LC-MS to create a fast and high-throughput screening test for CDG (Lacey et al 2001). A decade later, the emergence of advanced quadrupole time-of-flight (QTOF) detection in combination with nanoLC-ESI-MS enabled the development of high resolution intact transferrin glycoprofiling which in turn improved the CDG diagnostics (van Scherpenzeel et al 2015). Normal transferrin IEF profiles have been observed in some CDG-I and -II cases, such as ALG14-CDG, ALG11-CDG, MOGS-CDG, SLC35A3-CDG, and SLC35C1-CDG (Lefeber 2016; Al Teneiji et al 2017), as well as in some defects in sugar metabolism like GNE-CDG (Voermans et al 2010), NANS-CDG (Van Karnebeek et al 2016), PGM3-CDG (Stray-Pedersen et al 2014), and also in a tissue-specific and GA homeostasis defect of VPS13B-CDG/Cohen syndrome (Duplomb et al 2014).

Transferrin glycopeptide analysis, which was established by Wada et al in 2004 is important to reveal the structural information on both glycans and proteins. For example, the hybrid type glycan which is diagnostic for GA mannosidase defects of MAN1B1-CDG can only be found at Asn-432 but not on Asn-630 (Wada 2016). For O-glycan analysis, Wada et al developed plasma Apo C-III MALDI MS (Wada et al 2012).

For plasma glycomics, MALDI MS was used to analyze permethylated total plasma N-glycans (Guillard et al 2009), or combined plasma N-glycan and O-glycans MS (Xia et al 2013). MALDI MS was also suitable to analyze N-glycans released from fibroblast homogenates of SLC35A3 and healthy controls (Edvardson et al 2013), which is a useful alternative when transferrin glycosylation turned out to be normal. The patient cell homogenate showed a shift toward decreased branching of glycans compared to control, likely due to the lack of UDP-GlcNAc, which is a critical factor in the production of β1,6-branched (tetra-antennary) structures (Sasai et al 2002).

Both total plasma and intact transferrin glycoprofiling have their own advantages in the diagnosis of CDG. Recently, intact transferrin MS has successfully identified a series of novel CDG, such as PGM1-CDG (Tegtmeyer et al 2014) and MAN1B1-CDG (van Scherpenzeel et al 2014). A unique combination of CDG-I (lack of complete glycans) and CDG-II (truncated glycans especially lacking Gal residue) in PGM1-CDG is easily seen by intact transferrin glycoprofiling. Moreover, it is also used to follow-up the biochemical improvement of several patients that underwent an oral D-galactose supplementation (Voermans et al 2017, Wong et al 2017). It has become the primary diagnostic test for CDG, also including fast identification of B4GALT1-CDG, MGAT2-CDG, SLC35A1-CDG, and SLC35A2-CDG (van Scherpenzeel et al 2015).

However, there are several CDG types which cannot be discriminated by intact transferrin glycoprofiling which required total (released) plasma N-glycans for structural insights into the complete mixture of plasma glycoproteins. For example, for diagnosis of SLC35C1-CDG (GDP-fucose transporter defect), global N-glycan mapping is more suitable, because of its higher degree of fucosylation than intact transferrin (Guillard et al 2011). It is also able to detect the N-tetrasaccharide for ALG1-CDG diagnosis and highly abundant abnormal high mannose species of 3 Man- and 4 Man-glycans in PMM2-CDG and MPI-CDG (Zhang et al 2016), and to show abnormal profiles for tissue-specific glycosylation defects for VPS13B-CDG, also called Cohen syndrome (Duplomb et al 2014). In the case of MOGS-CDG which showed a normal transferrin profile, accumulation of several high mannose species from total IgG N-glycans were detected by plasma glycomics (Sadat et al 2014). In summary, Table 1 provides a detailed overview of the glycoprofiling data of total plasma N-glycans and intact transferrin for diagnosis of different CDG subtypes.

**Table 1:**
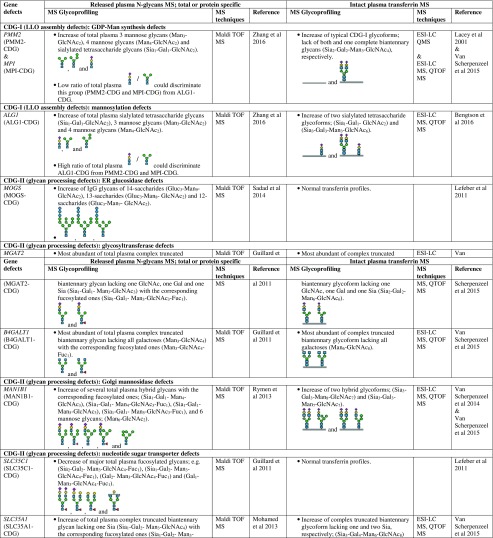

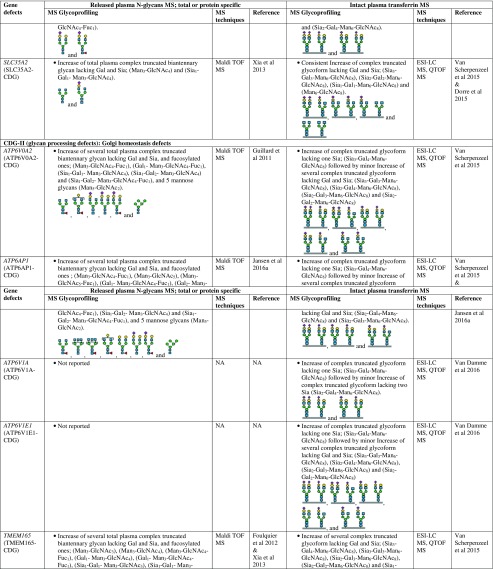

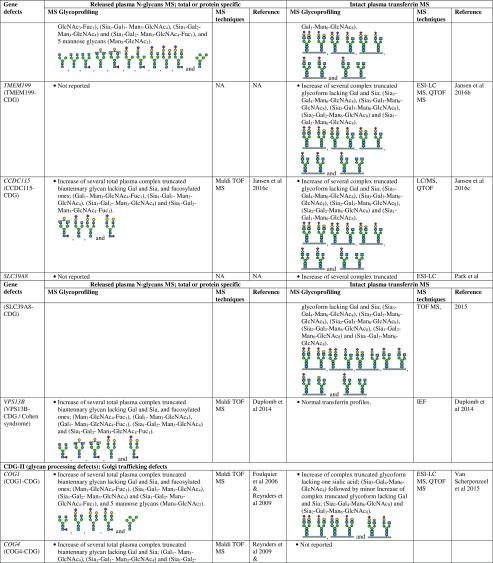

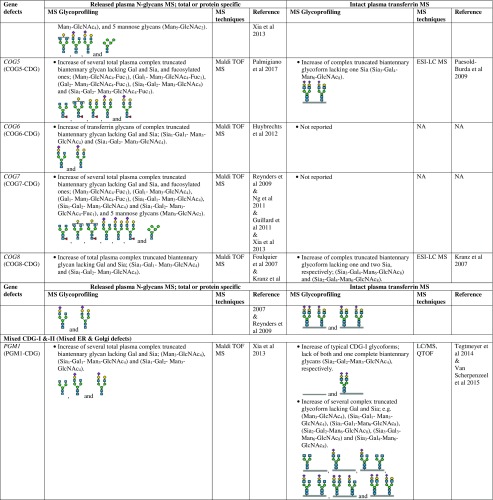
Overview of plasma glycosylation features from total (released) N-glycans and intact transferrin MS profiling for CDG diagnosis

## Overview of the current CDG diagnostic workflow

**Fig. 2:**
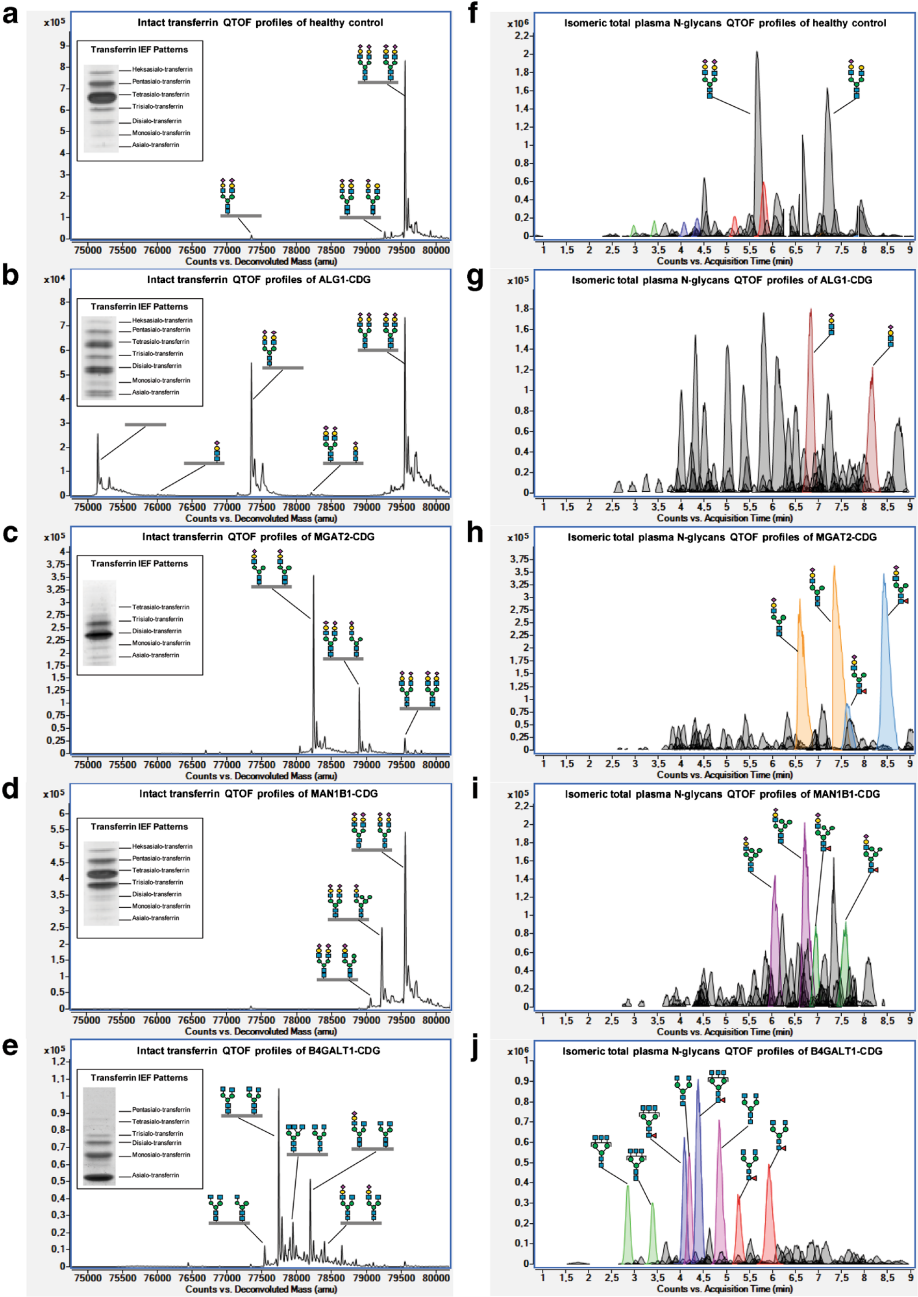
Intact transferrin IEF and MS profiles, and total plasma N-glycans MS profiles in healthy control, ALG1-CDG (cdg-Ik), MGAT2-CDG (cdg-IIa), MAN1B1-CDG, and B4GALT1-CDG (cdg-IIb). Deconvoluted QTOF mass spectrum of intact transferrin showed a high intensity of the peak indicating: (a) two bi-antennary fully sialylated (complete) glycoforms (79,557 Da) corresponding with the major presence of tetrasialo-transferrin bands on IEF patterns in healthy control; (b) lack of both (75,146 Da) and one (77,351 Da) complete glycoforms (CDG-I signatures) corresponding with the major increase of asialo- and disialo-transferrin bands on IEF patterns, as well as two minor peaks indicating the N-tetrasaccharide (76,007 & 78,212 Da) in ALG1-CDG; (c) two truncated glycoforms lacking N-acetylglucosamine (78,243 & 78,900 Da) corresponding with the major increase of trisialo- and disialo-transferrin bands on IEF patterns in MGAT2-CDG; (d) two hybrid glycoforms (79,059 & 79,221 Da) corresponding with the major increase of trisialo-transferrin bands on IEF patterns in MAN1B1-CDG; (e) five truncated glycoform lacking galactose (77,541, 77,743, 77,947, 78,196, & 78,399 Da), which are mostly non-sialylated species, corresponding with the major increase of asialo- and monosialo-transferrin on IEF patterns (mimicking CDG-I profiles) in B4GALT1-CDG. Extracted compound chromatrograms (ECCs) of isomeric native total plasma N-glycans profiling showed the most abundant compound peaks indicating: (f) di- and mono-sialylated bi-antennary N-glycans (5.6 & 7.2 min, respectively) in healthy control; (g) N-tetrasaccharide (6.8 & 8.2 min) in ALG1-CDG; (h) mono-sialylated N-glycans lacking N-acetylglucosamine (6.6 & 7.4 min) and its fucosylated species (7.6 & 8.3 min) in MGAT2-CDG; (i) hybrid N-glycans (6.1 & 6.7 min) and its fucosylated species (7.0 & 7.6 min) in MAN1B1-CDG; (j) two non-sialylated N-glycans lacking galactose, tri-antenna (2.9 & 3.4 min), and bi-antenna (4.2 & 4.9 min), and its fucosylated species; tri-antennary (4.1 & 4.4 min) and bi-antennary glycans (5.3 & 5.9 min) in B4GALT1-CDG

As one of the most abundant glycoproteins in human plasma, transferrin has been used traditionally as a biomarker for N-glycosylation defects. Plasma transferrin isoelectric focusing (IEF) is recognized as the classical laboratory method (Jaeken et al 1984) and widely used as a routine screening test for CDG. Human transferrin has two glycosylation sites on Asn-432 and Asn-630, carrying both a bi-antennary glycan with two terminal, negatively charged sialic acid residues. In normal conditions, most transferrin molecules consist of a total of four sialic acid residues which are displayed as a single major band of tetrasialo-transferrin in the IEF pattern (Fig. 2a). The CDG-I IEF pattern shows an increased band of disialo-transferrin and asialo-transferrin, owing to the occurrence of transferrin isoforms with two sialic acids and no sialic acid, respectively (Fig. 2b). CDG-II IEF patterns show high variability as depicted for MGAT2-CDG (Fig. 2c), MAN1B1-CDG (Fig. 2d), and B4GALT1-CDG (Fig. 2e), with increased trisialo-transferrin or increased asialo- and monosialo-transferrin, respectively. Untreated hereditary fructosemia and galactosemia, alcohol abuse, hepatopathy, and bacterial sialidases are known as secondary causes of abnormal transferrin glycosylation.

The diagnostics follow-up for CDG-I profiles started with an enzyme assay in fibroblasts or leukocytes for diagnosis of PMM2-CDG and PMI-CDG. If negative, the next step was to perform lipid-linked oligosaccharides (LLO) in fibroblasts (Gao and Lehrman 2002), or recently this has been replaced by WES using a filter for CDG-I genes and targeted sequencing of a CDG-I panel (Timal et al 2012). The recent discovery of a novel sialylated N-tetrasaccharide for ALG1-CDG and abnormal small high mannose glycan structures in PMM2-CDG and MPI-CDG has proved plasma N-glycan analysis to also be relevant for CDG-I diagnosis. Hence, plasma glycomics is highly useful especially for ALG1-CDG, since it is challenging to identify the defect by genetics due to 14 pseudo genes that can complicate the analysis (Zhang et al 2016). Another upcoming diagnostic choice is applying WES directly and Sanger sequencing to confirm the gene defect, but up till now it takes more time to result and is more expensive than conventional CDG screening (Van Scherpenzeel et al 2016).

The diagnostic follow-up approach for CDG-II profiles is an IEF assay of Apo C-III to profile the mucin type O-glycans (Wopereis et al 2003), to distinguish between an exclusive N-glycosylation defect and a combined disorder of N- and O-glycosylation (Wopereis et al 2007). Apo C-III has only a single O-glycan on Thr-74 which is terminally modified by up to two sialic acids to generate three main IEF isoforms: Apo C-III_0_ (no sialic acid), Apo C-III_1_ (one sialic acid), and Apo C-III_2_ (two sialic acids). Decreased sialylation on Apo C-III profiles has been reported in conserved oligomeric Golgi (COG) defects (Spaapen et al 2005; Foulquier et al 2006; Foulquier et al 2007; Kranz et al 2007; Morava et al 2007; Wopereis et al 2007; Zeevaert et al 2008; Ng et al 2011; Palmigiano et al 2017) and autosomal recessive cutis laxa type-2 (ARCL2) due to ATP6V0A2 dysfunctions (Morava et al 2005; Kornak et al 2008). The limitation of Apo C-III IEF is that it is not able to differentiate between the three possible Apo C-III_0_ isoforms; the “real unglycosylated Apo C-III” and Apo C-III with two non-sialylated monosaccharides namely Gal and GalNAc. They can easily be separated by Apo C-III MALDI MS (Wada 2016).

**Fig. 3:**
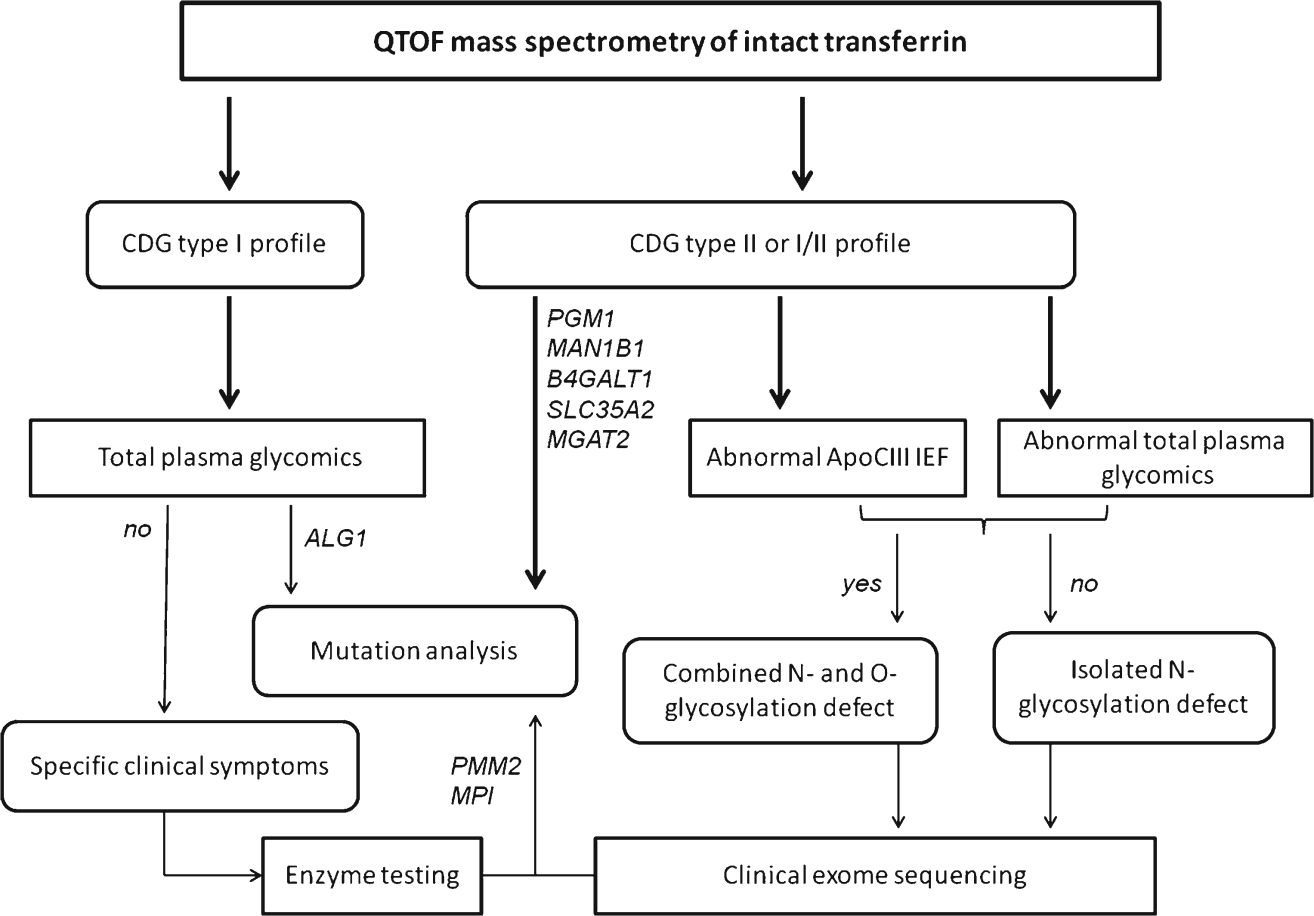
Clinical diagnostic flowchart for CDG. The combination of mass spectrometry with clinical exome sequencing and clinical phenotyping allows facile identification of the majority of known CDG-I and CDG-II subtypes in a diagnostic setting. The gene defects mentioned here are examples of characteristic diagnostic glycomics profiles in our laboratory. Please refer to Jaeken and Peanne (2017) and Peanne et al (2017) for clinical symptoms in CDG

So for more structural insight in glycans especially in CDG-II patients, MS is always the method of choice. This rapid profiling of abnormal glycans can be linked to potential gene defects based on the knowledge of glycosylation pathways. For example (Fig. 2): Detection of N-tetrasaccharide glycans in ALG1-CDG might be explained by the modification (galactosylation and sialylation) of the chitobiose glycan core, which accumulates due to cytosolic mannosyltransferase defects; Accumulation of truncated N-glycans lacking GlcNAc in MGAT2-CDG is likely due to the N-acetylglucosaminyltransferase defects in GA; Accumulation of truncated N-glycans lacking Gal could be due to B4GALT1 gene defect, encoding for galactosyltransferase in GA. Accumulation of hybrid N-glycans in MAN1B1-CDG is due to the ER mannosyltransferase defects. The structural information is important to narrow down the number of candidate genes, thereby greatly minimizing the time to diagnosis. The complete diagnostic workflow as described above is depicted in Fig. 3.

## Outlook

Based on the identification of several new genetic defects over the past four years by a combination of high-resolution intact transferrin analysis with WES, it might be concluded that clinical glycomics and NGS technology work in synergy by reducing the number of candidate genes and turnaround time to identify a CDG subtype. This approach creates opportunities to identify new genetic defects, but beyond this observation, glycomics has the benefit that the data do not only reflect the genes, but also environmental influences, which create possibilities for therapy monitoring and intervention. The first example was PGM1-CDG (Voermans et al 2017), but nowadays, several new genetic defects appear to be involved in sugar metabolism. Surprisingly, not all these genetic defects show abnormal transferrin glycosylation. Examples are NANS, GNE, PGM3, and Cohen syndrome. NANS and GNE are genetic defects in the sialic acid biosynthesis pathway, and Cohen syndrome patients have a mutation in *VPS13B*, a protein important for proper GA function. It is known that there are tissue-specific glycosylation processes, for example gamma-glutamyl transpeptidase synthetized by the liver or the kidney (West et al 2010), and that there will be other proteins than transferrin which do show abnormal glycosylation, e.g., muscle proteins for GNE patients.

Native glycopeptide profiling would be an attractive method to obtain insight into protein-specific glycosylation. For some isolated proteins like human transferrin, human alpha-1-acid glycoprotein, influenza A virus hemagglutinin, and human IgG, this has been performed (Khatri et al 2014). If this could work for highly complex protein mixtures like serum or plasma (Zielinska et al 2010), an enormous amount of data would show up and provide us with new mechanistic insights into tissue-specific glycosylation and likely yield several glycoprotein biomarkers for CDG and also common disorders (Yang et al 2017). Knowledge about the human glycoproteome is still very limited, but this new system-wide mapping technology will allow the study of fundamental questions in glycobiology, such as dynamics, macro- and microheterogeneity, tissue-specific glycosylation, and the function of certain glycans in specific biological contexts (Thaysen-Andersen and Packer 2014). In this context, genetic deficiencies beyond the classical CDG, which are in O-glycosylation, e.g., in O-GalNAc, O-mannose, and O-fucose glycosylation or in glycolipid or glycosaminoglycan biosynthesis (comprehensive review in Hennet 2012), have to be mentioned. There is no general diagnostic test available for these rare genetic disorders because of the structural heterogeneity of O-glycans and their tissue-specific expression. Because of the advancement in genetics and bioinformatics, we foresee glycomics becoming interlinked with metabolomics and proteomics, thereby opening research avenues to unravel protein glycosylation in a tissue- or cell-specific manner, to understand the biochemical mechanisms of glycosylation, and to ultimately develop or improve new (sugar based) therapies for this group of so far mainly untreatable disorders.

Another advance in technology is intact protein profiling. Both in the chromatographic part as well as the data analysis there are recent developments which enable the analysis of protein mixtures to have multiple biomarkers in one assay. A large number of biomarkers used in clinical laboratories are glycopeptides of which variations in glycosylation are not taken into account, and only protein expression levels are measured. Examples are alpha-1-antitrypsin for chronic obstructive pulmonary disease (COPD), haptoglobin for gastric cancer, and human chorionic gonadotrophin for ovarian and testicular tumors (Van Scherpenzeel et al 2016). For two intact plasma glycoproteins, namely alpha-fetoprotein and transferrin, it is already known that it is essential to include glycosylation analysis for specificity and sensitivity of the marker. There is an enormous potential to improve first line diagnosis when the level of glycosylation is taken into account on top of the current protein expression levels. With the upcoming use of targeted mass spectrometry in clinical laboratories, the glycan part of the biomarker might easily be included to improve sensitivity and specificity of the marker without additional time-to-result.

With the example of effective D-galactose supplementation on the improvement of glycosylation in PGM1-CDG, which was derived from the intact transferrin glycosylation profile (Tegtmeyer et al 2014), there is emerging interest in the application of sugars as supplemental therapies for metabolic disorders or as supportive therapy to improve the mechanism of action of known therapies, such as chemotherapy, for which 2-deoxyhexose was used to enhance the therapeutic effect by inhibiting glycolysis and even induce an effective antitumor immune response (Beneteau et al 2012). The way to obtain insight into these mechanisms will be one new glycomics area, in which the building blocks of glycosylation, comprising sugar-phosphates and nucleotide sugars, are analyzed. By studying the flux through sugar metabolism, dynamic insights will be obtained, which could lead to the development of new therapies and improve current ones. Bioinformatics will become increasingly important to extract the relevant information out of these big data sets, to visualize, and potentially integrate with other omics layers of information, for better understanding of the complex field of glycobiology.

## Abbreviations

2-AA: 2-aminobenzoic acid
2-AB: 2-aminobenzamide
2-AP: 2-aminopyridine
AFP-L3: fucosylated serum alpha-fetoprotein
Apo C-III: apolipoprotein C-III
ARCL2: autosomal recessive cutis laxa type-2
Asn: asparagine
CDG: congenital disorders of glycosylation
CDG-I: CDG type 1
CDG-II: CDG type 2
COG: conserved oligomeric Golgi
COPD: chronic obstructive pulmonary disease (COPD)
ER: endoplasmic Reticulum
ESI: electrospray Ionization
FDA: Food and Drug Administration
FT: Fourier transform
Fuc: fucose
GA: Golgi Apparatus
GAGs: glycosaminoglycans
Gal: galactose
GalNAc: *N*-acetylgalactosamine
Glc: glucose
GlcA: glucuronic acid
GlcNAc: *N*-acetylglucosamine
HILIC: hydrophilic interaction liquid chromatography
IEF: isoelectric focusing
LC: liquid chromatography
LLO: lipid-linked oligosaccharides
m/z: mass over charge ratio
MALDI: matrix assisted laser desorption ionization
Man: mannose
MS: mass spectrometry
NGS: next-generation sequencing
PGC: porous graphitized carbon
PNGaseF: *N*-glycosidase F
QTOF: quadrupole time-of-flight
Ser: serine
Sia: sialic acid
Thr: threonine
UV: ultraviolet
TOF: Time Of Flight
WES: whole exome sequencing
